# Supplementary material for: Combined Modality Therapy Based on Hybrid Gold Nanostars Coated with Temperature Sensitive Liposomes to Overcome Paclitaxel-Resistance in Hepatic Carcinoma
Source: Pharmaceutics. 2019 Dec 15;11(12):683. doi: 10.3390/pharmaceutics11120683 (PMC6969923; doi:10.3390/pharmaceutics11120683)
Supplement: Supplementary file 1 [file pharmaceutics-11-00683-s001.pdf]

pharmaceutics

MDPI

Supplementary Materials: Combined Modality Therapy Based on Hybrid Gold Nanostars Coated with Temperature Sensitive Liposomes to Overcome Paclitaxel-Resistance in Hepatic Carcinoma

Hongyan Zhu, Weili Han, Ye Gan, Qiaofeng Li, Xiaolan Li, Lanlan Shao, Dan Zhu and Hongwei Guo

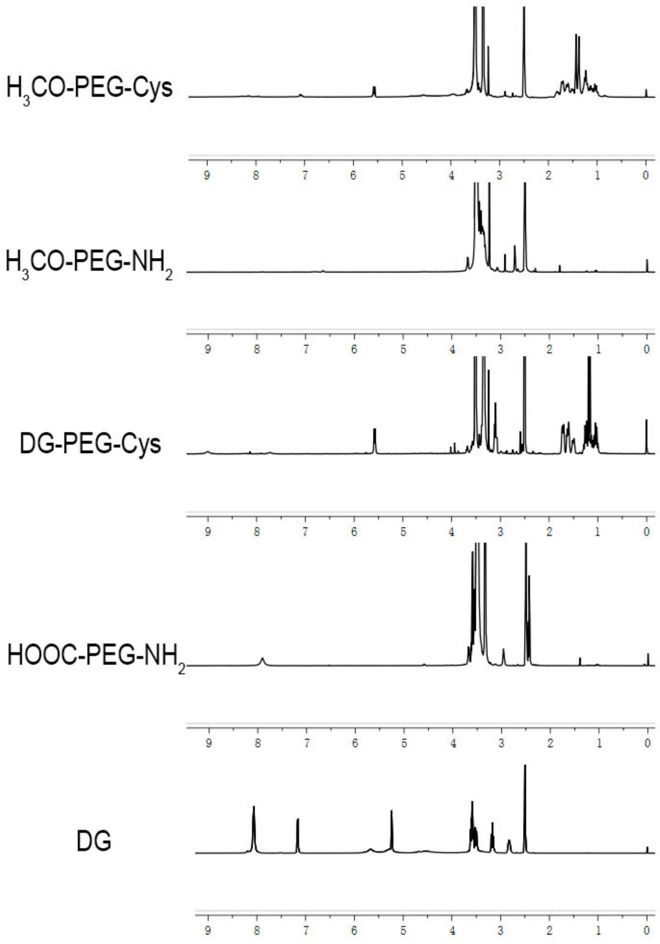

Figure S1. ^{1}HNMR spectra of DG, HOOC-PEG-NH$_2$, DG-PEG-Cys, H$_3$CO-PEG-NH$_2$, and H$_3$CO-PEG-Cys. DG: 2-Amino-2-deoxy-D-glucose; Cys: Cysteine.

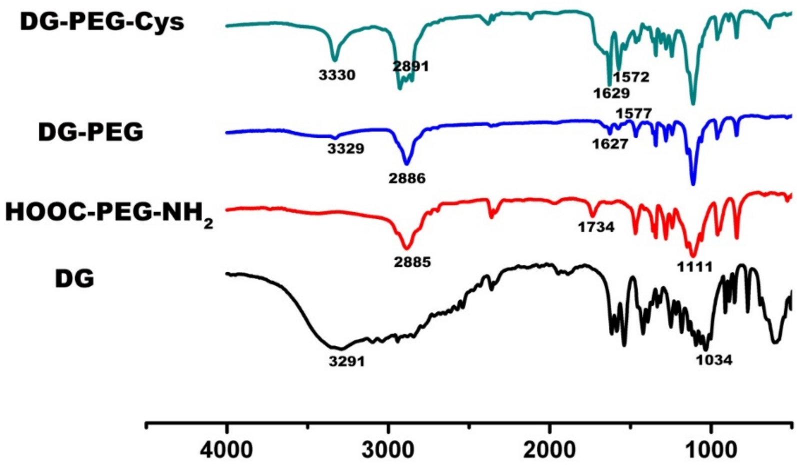

Figure S2. FTIR spectra of DG, HOOC-PEG-NH₂, DG-PEG and DG-PEG-Cys.

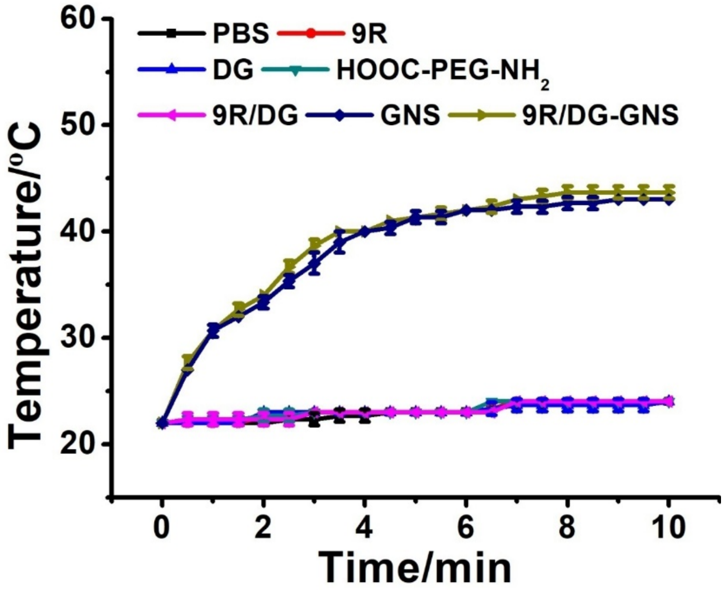

Figure S3. Temperature variation curves of 9R, DG, HOOC-PEG-NH2, 9R/DG, GNS and 9R/DG-GNS solution under the continuous NIR laser irradiation (808 nm, 0.5 W/cm2) for 10 min (n = 3).

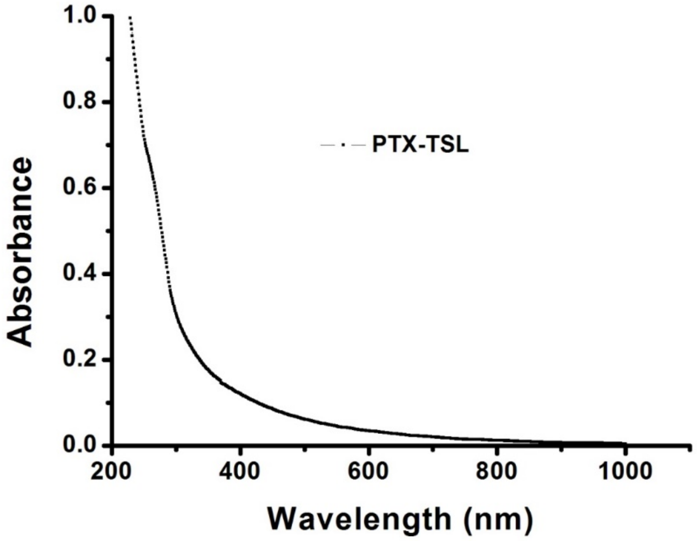

Figure S4. Ultraviolet absorption spectrum of PTX-TSL.
